# *REG Iα* gene expression is linked with the poor prognosis of lung adenocarcinoma and squamous cell carcinoma patients via discrete mechanisms

**DOI:** 10.3892/or.2013.2739

**Published:** 2013-09-19

**Authors:** MICHITAKA KIMURA, HIROSHI NAITO, TAKASHI TOJO, ASAKO ITAYA-HIRONAKA, YOSHIKO DOHI, MAMIKO YOSHIMURA, KAN-ICHI NAKAGAWARA, SHIN TAKASAWA, SHIGEKI TANIGUCHI

**Affiliations:** 1 Department of Thoracic and Cardiovascular Surgery, Nara Medical University, Kashihara, Nara 634-8522, Japan; 2 Department of Biochemistry, Nara Medical University, Kashihara, Nara 634-8522, Japan; 3 Nihon Gene Research Laboratories Inc., Sendai, Miyagi 983-0005, Japan

**Keywords:** *REG* family genes, *REG Iα* gene, lung cancer, prognostic factor, gene expression

## Abstract

The aim of the present study was to evaluate the effects of the *REG Iα* and *REG Iβ* genes on lung cancer cell lines, and thereafter, the expression of *REG* family genes (*REG Iα*, *REG Iβ*, *REG III*, *HIP/PAP* and *REG IV*) in lung cancer in relation to patient prognosis was evaluated. Lung adenocarcinoma (AD) and squamous cell carcinoma (SCC) cell lines expressing *REG Iα* or *REG Iβ* (HLC-1 REG Iα/Iβ and EBC-1 REG Iα/Iβ) were established, and cell number, cell invasive activity, and anchorage-independent cell growth were compared with these variables in the control cells. The expression levels of *REG* family genes were evaluated by real-time RT-PCR in surgically resected lung cancers, and disease-specific survival (DSS) curves were generated. The HLC-1 REG Iα/Iβ cell line showed significant increases in cell number and anchorage-independent cell growth compared with the control cells. EBC-1 REG Iα/Iβ cells showed significant increases in cell invasive activity and anchorage-independent cell growth as compared with the control cells. Except for the *REG Iβ* gene, expression of other *REG* family genes was observed in the surgically resected samples; however, DSS was significantly worse only in stage I patients who were positive for *REG Iα* expression than in patients who were negative for *REG Iα* expression. The effects of *REG Iα* on AD and SCC cells were different in the *in vitro* study, and a correlation between *REG Iα* expression and patient prognosis was noted in the *in vivo* study. Therefore, overexpression of *REG Iα* is a risk factor for poor prognosis caused by discrete mechanisms in AD and SCC patients.

## Introduction

In Japan, the number of lung cancer patients is increasing and lung cancer has become the leading and the second largest cause of cancer-related mortality in men and women, respectively (1). Since the improvement in diagnostic technologies for lung cancer, an increasing number of patients are being diagnosed in the early stages of the disease. In cases where non-small cell lung cancer (NSCLC) is diagnosed in the early stages, favorable prognoses have been reported after treatment with lobectomy (2–5), and lobectomy without any adjuvant therapy is an approved standard of therapy for these patients (3–5). However, we often encounter rapid tumor progression after lobectomy, even in these patients. If, therefore, the likelihood of this rapid progression could be predicted, it would be reasonable to initiate adjuvant therapy in advance.

The regenerating gene (*Reg*) was originally discovered in the regeneration of pancreatic β-cells (6–8). There are currently five genes in the *REG* family found in humans (*REG Iα*, *REG Iβ*, *REG III*, *HIP/PAP* and *REG IV*) (9), encoding a growth factor family of proteins involved not only in regeneration of damaged tissues but also in the growth of various types of cancers, including gastrointestinal cancer, cholangiocarcinoma, pancreatic cancer, breast cancer and prostate cancer (10–27). A correlation between *REG Iα* expression and poor prognosis has also been reported in NSCLC (28). While studies have indicated that poor prognosis in patients expressing *REG Iα* appears to be due to an increased cell number in gastric and pancreatic cancers (13,26), the impact of *REG Iα* on cancer cells has not been examined in NSCLC.

In the present study, the effects of the expression of *REG Iα* and *REG Iβ*, which has a similar structure to *REG Iα* and seems to have an identical function to *REG Iα*, on adenocarcinoma (AD) and squamous cell carcinoma (SCC) cells were examined *in vitro*. In addition, we investigated the correlation between expression of *REG* family genes and the prognosis of AD and SCC patients.

## Materials and methods

### Human lung cancer cell lines

The HLC-1 human lung adenocarcinoma cell line and the EBC-1 human squamous cell carcinoma cell line were obtained from Riken BioResource Center (Tsukuba, Japan). HLC-1 and EBC-1 cells were maintained in Ham’s F12 and minimum essential medium (MEM), respectively. No expression of any of the *REG* family genes was confirmed in these cells by real-time RT-PCR.

### Establishment of stable transfectants for REG Iα and REG Iβ

We established two cell lines expressing the *REG Iα* or the *REG Iβ* gene in HLC-1 and EBC-1 cells and one mock-transfected cell line as a control for each cell type. The expression vectors or a control vector (without insert DNA) were then transfected into HLC-1 or EBC-1 cells by electroporation (17). Stable transfectants were selected after 2 weeks of culture with 500 μg/ml Geneticin^®^ (Gibco, Carlsbad, CA, USA). *REG Iα* or *REG Iβ* expression was confirmed by real-time RT-PCR and immunoblot analysis of the culture medium, as previously described (17). The resulting Geneticin-resistant clones were designated as HLC-1 REG Iα-1, -2; HLC-1 REG Iβ-1, -2; HLC-1 mock; EBC-1 REG Iα-1, -2; EBC-1 REG Iβ-1, -2; and EBC-1 mock.

### Cell number, cell invasive capacity and anchorage-independent cell growth

For evaluation of cell growth in the HLC-1 and EBC-1 cell lines, cells were cultured in Ham’s F12 or MEM containing 1 or 0.5% FBS, respectively. The cell number for the HLC-1 cells was determined using a Cell Counting Kit-8 (Dojindo, Mashikimachi, Japan) on 1, 3, 5 and 7 days of culture, and that for EBC-1 was monitored on 0, 1, 2 and 3 days of culture. Increases in the cell number were expressed as the percentage of the cell number at culture day 1 or 0, respectively. Cell invasive activity was monitored using a Cultrex 96 Well BME Cell Invasion assay (Trevigen, Gaithersburg, MD, USA). To evaluate anchorage-independent cell growth, cells (1.75×10^3^) were plated into 12-well plates in culture medium containing 0.35% agar on top of 0.5% agar, prepared in the same medium. The plates were incubated at 37°C for 16 days. Colonies were stained with 0.005% crystal violet for 1 h. Colonies, containing at least 50 cells, were counted.

### Patients

Fifty-one AD and 23 SCC patients, who underwent surgery at Nara Medical University Hospital from 2004 to 2007, were enrolled. The present study was approved by the Ethics Committee of the Nara Medical University School of Medicine. Fifty-one were male and 23 were female, and the mean age was 68.3±1.1 years. Forty-six patients (AD, 32 patients; SCC, 14 patients) were in pathological stage I, 8 patients (AD, 2; SCC, 6) were in stage II and 20 patients (AD, 17; SCC, 3) were in stage III. Sixty-eight patients (AD, 47; SCC, 21) underwent complete resection and the remaining 6 patients (AD, 4; SCC, 2) in stage III received incomplete resection because of the extensive invasion of the tumors into the surrounding organs.

### Real-time RT-PCR of surgical tissue samples

Samples (tumor and normal lung tissues) were collected immediately after lung resection (surgical sample), and frozen in liquid nitrogen until RNA isolation. Total RNA was isolated for real-time reverse transcription-polymerase chain reaction (real-time RT-PCR), as previously described (27,28). The primers and probes (Table I) were synthesized by Nihon Gene Research Laboratories (Sendai, Japan). Real-time RT-PCR was then carried out using TaqMan^®^ Universal PCR Master Mix in an ABI PRISM^®^ 7700 Sequence Detection system (Applied Biosystems, Foster City, CA, USA). Expression of *REG* family genes was normalized with respect to β-actin. The cut-off levels for expression of each gene were set at the average + 3SD expression of the normal lung tissues. The expression of each *REG* family gene, which was higher or lower than the cut-off level, was defined as high or weak, respectively, and the absence of the expression of each gene was defined as no expression. For analysis of the correlation between the expression of each gene and prognosis, patients with high expression were defined as positive, and those with weak or absence of expression were defined as negative.

### Real-time RT-PCR of formalin-fixed paraffin-embedded (FFPE) samples

Total RNA was isolated from FFPE tissue specimens (AD, 10; SCC, 8, randomly selected) using the RNeasy FFPE kit (Qiagen, Hilden, Germany) and reverse transcribed as described above. Real-time PCR was performed using KAPA SYBR^®^ FAST qPCR Master Mix (Kapa Biosystems, Boston, MA, USA) and the Thermal Cycler Dice Real-Time System (Takara, Otsu, Japan) as previously described (29–31).

### Disease-specific survival

Patient death in the progression of lung cancer was defined as the end point. Kaplan-Meier survival curves for disease-specific survival (DSS) were constructed according to the expression of *REG Iα* or *REG IV* genes.

### Statistics

Data are expressed as the mean ± standard error of the mean (SEM), and cell number, cell invasive activity and anchorage-independent cell growth were compared by unpaired t-tests. Comparison of clinicopathological parameters according to the expression of the *REG Iα* gene was carried out by Chi-squared analyses. Kaplan-Meier survival curves for DSS were compared using the log-rank test. Correlations of the expression levels of the *REG Iα gene* from surgical and FFPE samples were analyzed using Pearson non-parametric tests. A P-value of <0.05 was considered to indicate a statistically significant result.

## Results

### Effects of the transfection of REG Iα and REG Iβ on cell number, cell invasive activity and anchorage-independent cell growth in lung cancer cells

**Figure 1:**
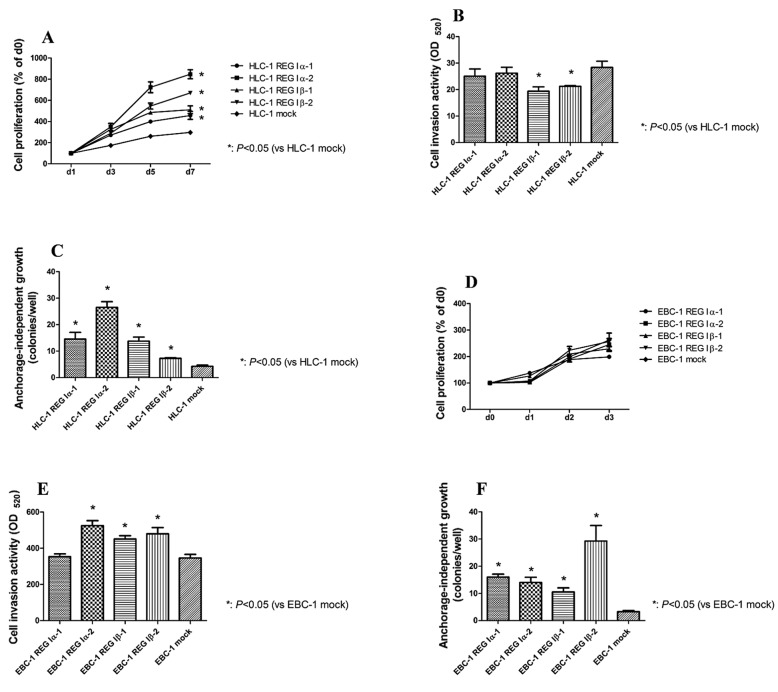
*REG Iα* and *Iβ* expression in lung adenocarcinoma (HLC-1) and lung squamous cell carcinoma (EBC-1) cells. Cells were stably transfected with *REG Iα* and *Iβ*. The effects of *REG Iα* or *Iβ* on proliferation (A and D), cell invasive activity (B and E), and anchorage-independent cell growth (C and F). HLC-1 and EBC-1 mock cells acted as mock-transfected controls.

The expression of *REG Iα* or *REG Iβ* in HLC-1 REG Iα/Iβ and EBC-1 REG Iα/Iβ cells was confirmed by real-time RT-PCR, whereas no expression of *REG Iα* or *REG Iβ* was detected in the HLC-1 and EBC-1 mock control cell lines. All of the HLC-1 REG Iα/Iβ-transfected cell lines showed a significant increase in cell number when compared with the HLC-1 mock cells on culture day 7 (Fig. 1A). In contrast, HLC-1 REG Iα cells did not show increased cell invasive activity when compared with the HLC-1 mock cells, while HLC-1 REG Iβ cells in fact showed a decelerated invasive potential (Fig. 1B). HLC-1 REG Iα/Iβ cells showed significant increases in anchorage-independent cell growth as compared with the HLC-1 mock cells (Fig. 1C).

By comparison, we observed no significant increases in cell number for the EBC-1 REG Iα/Iβ-transfected cells as compared with the EBC-1 mock cells after 3 days of culture (Fig. 1D). EBC-1 REG Iα-2, EBC-1 REG Iβ-1 and -2 cells showed increased cell invasive activity as compared with the EBC-1 mock cells (Fig. 1E), and all of the EBC-1 REG Iα/Iβ cell lines showed a significant increase in anchorage independent cell growth when compared with the EBC-1 mock cells (Fig. 1F).

### REG family gene expression in normal lung and tumor tissues

**Figure 2:**
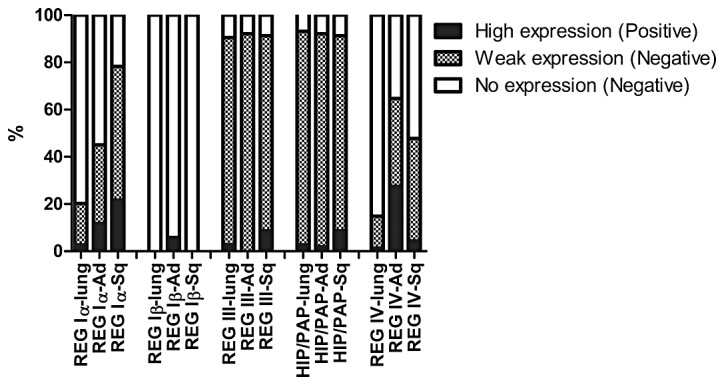
Expression levels of *REG* family genes in normal lung and tumor tissues. The cut-off levels for expression of each *REG* family gene were set at average + 3SD expression of the normal lung tissues. The expression of each *REG* family gene, which was higher or lower than the cut-off level, was defined as high expression (positive) or weak expression (negative), respectively. The absence of expression of each gene, was defined as no expression (negative). REG Iα-lung, *REG Iα* expression in normal lung tissues; REG Iα-Ad, *REG Iα* expression in AD; REG Iα-Sq, *REG Iα* expression in SCC; REG Iβ-lung, *REG Iβ* expression in normal lung tissues; REG Iβ-Ad, *REG Iβ* expression in AD; REG Iβ-Sq, *REG Iβ* expression in SCC; REG III-lung, *REG III* expression in normal lung tissues; REG III-Ad, *REG III* expression in AD; REG III-Sq, *REG III* expression in SCC; HIP/PAP-lung, *HIP/PAP* expression in normal lung tissues; HIP/PAP-Ad, *HIP/PAP* expression in AD; HIP/PAP-Sq, *HIP/PAP* expression in SCC; REG IV-lung, *REG IV* expression in one normal lung tissue; REG IV-Ad, *REG IV* expression in AD; REG IV-Sq, *REG IV* expression in SCC.

Expression of all the *REG* family genes, except for *REG Iβ*, was observed in both normal lung and tumor tissues (Fig. 2). *REG Iβ* was expressed only in 3 AD patients. The expression of *REG III* and *HIP/PAP* was noted in ~90% of both normal lung and tumor tissues. The expression profile of these genes was not different between the normal lung and tumor tissues. Comparatively, *REG Iα* and *REG IV* mRNAs were observed more frequently in tumor tissues than in normal lung tissues. Therefore, we focused on the correlation between the expression of *REG Iα* and *REG VI* in tumor tissues and the prognosis of patients in the subsequent studies.

### REG Iα expression and prognosis of patients

**Figure 3:**
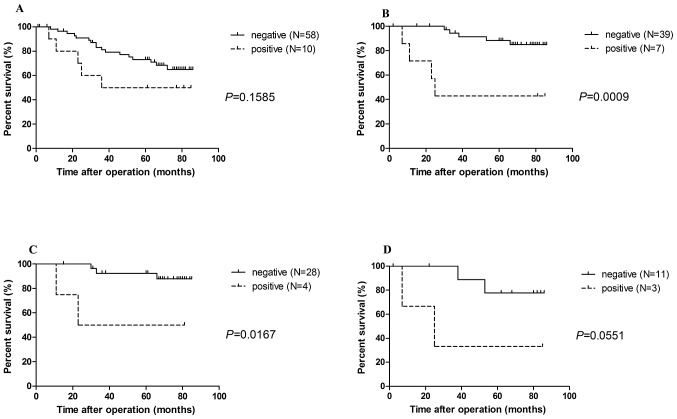
Disease-specific survival of patients with positive and negative expression of the *REG Iα* gene. Patient death by progression of lung cancer (disease-specific survival; DSS) was defined as the end point. DSS of patients with positive and negative expression for *REG Iα* in (A) all patients, (B) stage I patients, (C) stage I adenocarcinoma patients and (D) stage I squamous cell carcinoma patients.

In the 68 patients (AD, 47; SCC, 21) who underwent complete resection, there were no significant differences in gender, age or pathological stage between patients who were positive and those who were negative for *REG Iα* expression (Table II). First, we evaluated the relationship between the expression of *REG Iα* and prognosis in these 68 patients. Ten patients (AD, 5; SCC, 5) showed positive expression for *REG Iα*, whereas 58 patients (AD, 42; SCC, 16) showed negative expression. Overall, there was no significant correlation between patients with positive or negative *REG Iα* expression and prognosis (P=0.1585; Fig. 3A). However, when we examined the 46 stage I patients separately, we observed a significantly worse prognosis in patients with positive *REG Iα* expression (n=7) than those with negative *REG Iα* expression (n=39) (P=0.0009; Fig. 3B). In addition, the 5-year survival in these patients with positive *REG Iα* expression was significantly lower than that in patients with negative *REG Iα* expression (42.9 vs. 84.9%; P=0.034). Next, we divided 46 stage I patients into two groups by histological types: AD (n=32) and SCC (n=14). The prognosis of stage I AD patients positive for *REG Iα* expression was significantly worse than that for patients negative for *REG Iα* (P=0.0167; Fig. 3C). In stage I SCC patients, however, there was a trend toward poor prognosis in patients with positive *REG Iα* expression (P=0.0551; Fig. 3D) when compared with the negative patients. Concerning *REG IV* expression and patient prognosis, no correlation was noted for any of the subgroupings detailed above (data not shown).

### REG Iα expression in FFPE samples

**Figure 4:**
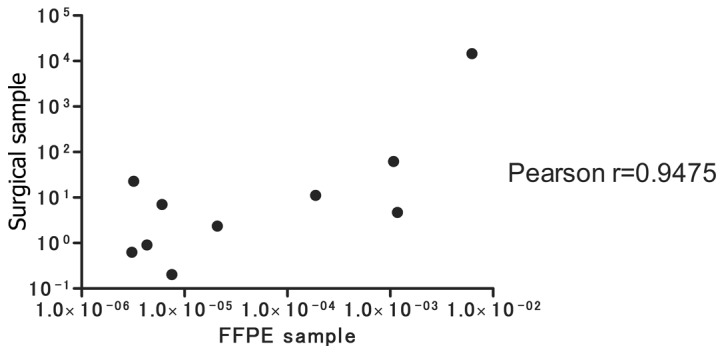
Correlation of the expression of *REG Iα* from surgical and FFPE samples. A significant correlation was noted between the expression of *REG Iα* in surgical and FFPE samples (Pearson r=0.9475).

Next, we tested *REG Iα* expression in FFPE samples taken from a random selection of AD (n=10) and SCC (n=8) patients, and compared the results with *REG Iα* expression in surgical samples. As shown in Fig. 4, a significant correlation was noted between *REG Iα* expression from the surgical samples and that from the FFPE samples (Pearson correlation r=0.9475).

## Discussion

The effect of *REG* family genes on malignancies has been studied mainly in gastrointestinal cancers (10–15,17–20). *REG Iα* and *REG IV* was found to be correlated with poor prognosis in gastric and colorectal cancers (12–15,27). A correlation, however, has recently been reported to exist between high expression of *REG Iα* and a more favorable prognosis in esophageal cancer patients (18). The authors indicated that high expression of *REG Iα* enhanced the chemosensitivity and radiosensitivity of esophageal cancer cells, which may explain the better prognosis of the patients (17). This high expression is contradictory to the findings of others (14–16,26–28), but may be explained by histopathological differences between other gastrointestinal tumors and esophageal tumors; most esophageal cancers are SCCs, whereas other gastrointestinal cancers are ADs. Gastric AD patients with *REG Iα* expression were reported to show poor prognosis, and REG Iα-expressing cells exhibit an increase in cell number (13). In lung cancer, the reason why *REG Iα* expression leads to a poorer prognosis has not been clarified (28). We hypothesized that discrete mechanisms may exist in lung cancer cells due to histological distinctions between AD and SCC cells. Thus, we performed an *in vitro* study to clarify the effect of the expression of *REG Iα* and *REG Iβ*, which has a similar structure to *REG Iα* and seems to have an identical function to *REG Iα*, on AD and SCC cells.

In AD cells, both *REG Iα* and *Iβ* increased cell numbers as compared with the control cells, whereas, in SCC cells, neither *REG Iα* nor *Iβ* influenced cell number. In contrast, no clear effect was found in the AD cells in regards to enhanced cell invasion in response to either gene, whereas a positive effect was demonstrated in SCC cells. Anchorage-independent cell growth, however, was upregulated for both cell types expressing *REG Iα* and *Iβ*. These results suggest that the effect of *REG Iα* and *Iβ* on lung cancer is specific in regards to the type of tumor. From these results, we hypothesized that patients who express the *REG Iα* and *Iβ* genes may have poor prognosis by different mechanisms as described above. We evaluated the relationship between the expression of these genes and patient prognosis.

Despite recent findings that a link exists between the *REG* family genes and various significant cancer subtypes (10–27), including lung cancer (28), the expression levels of this family of genes have not been explored. In the present study, we evaluated the expression levels of *REG* family genes in lung cancer tissues. Almost all of the *REG* family genes were expressed both in normal lung and tumor tissues except for *REG Iβ*. However, positive ratios of gene expression levels varied for each *REG* family member. *REG III* and *HIP/PAP* were high in both normal lung and tumor tissues. Conversely, the expression ratios of *REG Iα* and *REG IV* in tumor tissues were higher than those in normal lung tissues. Previous studies have shown that prognoses are worse in patients with stomach, pancreatic, lung, and breast cancers with high *REG Iα* expression (14–16,26–28). Likewise, high *REG IV* expression in colorectal and prostate cancers is linked with a worse prognosis (12,24). Therefore, we also tested correlations between the expression of *REG Iα* and *REG IV* and patient prognosis in lung cancer patients. We found that a high expression of *REG Iα* was correlated with poor prognosis in stage I lung cancer patients, suggesting that *REG Iα* is a reliable marker for the prognosis of stage I lung cancer patients. The *in vitro* study confirmed that the *REG Iβ* gene promoted an increased cell number and anchorage-independent cell growth in AD cells, and increased cell invasive activity and anchorage-independent cell growth in SCC cells. However, *REG Iβ* was expressed only in 3 AD patients. Therefore, the expression of the *REG Iβ* gene seems to have no meaning clinically. Together with the *in vitro* data, we surmised that poorer prognosis in *REG Iα*-expressing AD patients stems from an increase in cell number and anchorage-independent cell growth, whereas the tendency for a poorer prognosis in SCC patients with positive expression of *REG Iα* might be due to enhanced cell invasion and anchorage-independent cell growth. In comparison, we found no correlation between *REG IV* and prognosis, suggesting a different role for *REG IV* in lung cancer (12,24).

As it is not easy to obtain fresh frozen surgical samples, we also evaluated the expression of the *REG Iα* gene in FFPE samples to compare an easier, more practical and more economical method for RNA extraction for future clinical applications. We found a significant correlation in *REG Iα* expression between the two different sampling and real-time RT-PCR methods (Fig. 4). Clinically, the positive effect of *REG Iα* in lung cancer cells implies that *REG Iα* could be used as an indicator to initiate adjuvant therapy, even in stage I lung cancer patients; alternatively, it may become a target for therapy or a marker of chemosensitivity and radiosensitivity (18).

One of the limitations of the present study was the small number of participants in the SCC group, as the correlation between *REG Iα* and prognosis could only be evaluated in 14 stage I SCC patients. This may explain the lack of a significant correlation between the expression of *REG Iα* and SCC prognosis.

In summary, the *REG Iα* gene increased the cell number and anchorage-independent cell growth of lung adenocarcinoma cells, and the cell invasive activity and anchorage-independent cell growth in lung squamous cell carcinoma. Overexpression of the *REG Iα* gene is a risk factor for poor prognosis in lung cancer patients functioning via different mechanisms in adenocarcinoma and squamous cell carcinoma.

## Figures and Tables

**Table I:** Primers and probes for real-time RT-PCR.

Gene (Accession no.)	Sequence
β-actin (NM_001101)	Forward: 5′-GCGAGAAGATGACCCAGA-3′ Reverse: 5′-CAGAGGCGTACAGGGATA-3′ Probe: 5′-FAM-ACAGCCTGGATAGCAACGTACATGGCT-TAMRA-3′
REG Iα (NM_002909)	Forward: 5′-AGGAGAGTGGCACTGATGACTT-3′ Reverse: 5′-TAGGAGACCAGGGACCCACTG-3′ Probe: 5′-FAM-TGGCCTCCATGACCCCAAAAAGAAC-TAMRA-3′
REG Iβ (NM_006507)	Forward: 5′-GCTGATCTCCTCCCTGATGTTC-3′ Reverse: 5′-GGCAGCTGATTCGGGGATTA-3′ Probe: 5′-FAM-TGTCTCTGAGCCAAGGCCAGGAGTCCCA-TAMRA-3′
REG III (AB161037)	Forward: 5′-GAATATTCTCCCCAAACTG-3′ Reverse: 5′-GAGAAAAGCCTGAAATGAAG-3′ Probe: 5′-FAM-CCTACCTGACTACCTTGTCATGATCCTCC-TAMRA-3′
HIP/PAP (NM_138937)	Forward: 5′-AGAGAATATTCGCTTAATTCC-3′ Reverse: 5′-AATGAAGAGACTGAAATGACA-3′ Probe: 5′-FAM-CCAACCTGACCACCTCATTCTTATCTTTC-TAMRA-3′
REG IV (AY007243)	Forward: 5′-ATCCTGGTCTGGCAAGTC-3′ Reverse: 5′-CGTTGCTGCTCCAAGTTA-3′ Probe: 5′-FAM-CTGTGCTGAGATGAGCTCCAATAACAACTT-TAMRA-3′

**Table II:** Characteristics of the lung cancer patients with complete resection.

	Adenocarcinoma			Squamous cell carcinoma		
		
	*REG Iα* gene expression			*REG Iα* gene expression		
		
	Positive	Negative	P-value	Positive	Negative	P-value
Gender						
Male	3	25	0.64	4	15	0.97
Female	2	17		1	1	
Age (years)	72.0±2.2	66.0±1.5	0.15	73.0±2.7	71.6±2.0	0.72
Tumor stage						
I	4	28	0.92	3	11	0.86
II and III	1	14		2	5	

## Abbreviations

AD: adenocarcinoma
DSS: disease-specific survival
FFPE: formalin-fixed paraffin-embedded
NSCLC: non-small cell lung cancer
*Reg*: regenerating gene
SCC: squamous cell carcinoma

## References

[1] (2008). Cancer statistics in Japan ’08.

[2] Whitson BA, Groth SS, Duval SJ, Swanson SJ, Maddaus MA (2008). Surgery for early-stage non-small cell lung cancer: a systematic review of the video-assisted thoracoscopic surgery versus thoracotomy approaches to lobectomy. Ann Thorac Surg.

[3] Wright G, Manser RL, Byrnes G, Hart D, Campbell DA (2006). Surgery for non-small cell lung cancer: systematic review and meta-analysis of randomised controlled trials. Thorax.

[4] Mountain CF (1997). Revisions in the International System for Staging Lung Cancer. Chest.

[5] Ginsberg RJ, Rubinstein LV (1995). Randomized trial of lobectomy versus limited resection for T1 N0 non-small cell lung cancer. Lung Cancer Study Group Ann Thorac Surg.

[6] Terazono K, Yamamoto H, Takasawa S (1988). A novel gene activated in regenerating islets. J Biol Chem.

[7] Watanabe T, Yonemura Y, Yonekura H (1994). Pancreatic beta-cell replication and amelioration of surgical diabetes by Reg protein. Proc Natl Acad Sci USA.

[8] Takasawa S, Ikeda T, Akiyama T (2006). Cyclin D1 activation through ATF-2 in Reg-induced pancreatic β-cell regeneration. FEBS Lett.

[9] Nata K, Liu Y, Xu L (2004). Molecular cloning, expression and chromosomal localization of a novel human *REG* family gene, *REG III*. Gene.

[10] Sekikawa A, Fukui H, Fujii S (2005). REG Iα protein may function as a trophic and/or anti-apoptotic factor in the development of gastric cancer. Gastroenterology.

[11] Mitani Y, Oue N, Matsumura S (2007). Reg IV is a serum biomarker for gastric cancer patients and predicts response to 5-fluorouracil-based chemotherapy. Oncogene.

[12] Oue N, Kuniyasu H, Noguchi T (2007). Serum concentration of Reg IV in patients with colorectal cancer: overexpression and high serum levels of Reg IV are associated with liver metastasis. Oncology.

[13] Fukui H, Fujii S, Takeda J (2004). Expression of REG Iα protein in human gastric cancers. Digestion.

[14] Yamagishi H, Fukui H, Sekikawa A (2009). Expression profile of REG family proteins REG Iα and REG IV in advanced gastric cancer: comparison with mucin phenotype and prognostic markers. Mod Pathol.

[15] Dhar DK, Udagawa J, Ishihara S (2004). Expression of regenerating gene I in gastric adenocarcinomas: correlation with tumor differentiation status and patient survival. Cancer.

[16] Sasaki Y, Minamiya Y, Takahashi N (2008). REG1A expression is an independent factor predictive of poor prognosis in patients with breast cancer. Ann Surg Oncol.

[17] Hayashi K, Motoyama S, Koyota S (2008). REG I enhances chemo- and radiosensitivity in squamous cell esophageal cancer cells. Cancer Sci.

[18] Hayashi K, Motoyama S, Sugiyama T (2008). REG Iα is a reliable marker of chemoradiosensitivity in squamous cell esophageal cancer patients. Ann Surg Oncol.

[19] Motoyama S, Sugiyama T, Ueno Y (2006). REG I expression predicts long-term survival among locally advanced thoracic squamous cell esophageal cancer patients treated with neoadjuvant chemoradiotherapy followed by esophagectomy. Ann Surg Oncol.

[20] Usami S, Motoyama S, Koyota S (2010). Regenerating gene I regulates interleukin-6 production in squamous esophageal cancer cells. Biochem Biophys Res Commun.

[21] Kiji T, Dohi Y, Takasawa S, Okamoto H, Nonomura A, Taniguchi S (2005). Activation of regenerating gene *Reg* in rat and human hearts in response to acute stress. Am J Physiol Heart Circ Physiol.

[22] Kiji T, Dohi Y, Nishizaki K (2003). Enhancement of cell viability in cryopreserved rat vascular grafts by administration of regenerating gene (REG) inducers. J Vasc Res.

[23] Harada K, Zen Y, Kanemori Y (2001). Human REG I gene is up-regulated in intrahepatic cholangiocarcinoma and its precursor lesions. Hepatology.

[24] Ohara S, Oue N, Matsubara A (2008). Reg IV is an independent prognostic factor for relapse in patients with clinically localized prostate cancer. Cancer Sci.

[25] Zhou L, Zhang R, Wang L (2010). Upregulation of REG Iα accelerates tumor progression in pancreatic cancer with diabetes. Int J Cancer.

[26] Yonemura Y, Sakurai S, Yamamoto H (2003). *REG* gene expression is associated with the infiltrating growth of gastric carcinoma. Cancer.

[27] Zheng HC, Sugawara A, Okamoto H (2011). Expression profile of the *REG* gene family in colorectal carcinoma. J Histochem Cytochem.

[28] Minamiya Y, Kawai H, Saito H (2008). REG1A expression is an independent factor predictive of poor prognosis in patients with non-small cell lung cancer. Lung Cancer.

[29] Takasawa S, Kuroki M, Nata K (2010). A novel ryanodine receptor expressed in pancreatic islets by alternative splicing from type 2 ryanodine receptor gene. Biochem Biophys Res Commun.

[30] Ota H, Tamaki S, Itaya-Hironaka A (2012). Attenuation of glucose-induced insulin secretion by intermittent hypoxia via down-regulation of CD38. Life Sci.

[31] Masui T, Ota I, Itaya-Hironaka A (2013). Expression of REG III and prognosis in head and neck cancer. Oncol Rep.

